# Chain Reduction of CHCl_3_ Photocatalyzed by SPEEK/PVA Films Swollen in Air-Saturated HCO_2_Na Solutions

**DOI:** 10.3390/ma16206629

**Published:** 2023-10-10

**Authors:** Radini Dissanayaka, Md Safiqul Islam, G. Mills

**Affiliations:** 1 Department of Chemistry & Biochem, Auburn University, Auburn, AL 36849, USA; rhd0004@auburn.edu; 2 Department of Chemistry, University of Dhaka, Dhaka 1000, Bangladesh; safique@du.ac.bd

**Keywords:** photoreduction, photocatalyst, CHCl_3_, chain reaction, SPEEK, cross-linked polymer film, polyelectrolyte

## Abstract

Thin cross-linked films containing sulfonated poly(ether etherketone), SPEEK, and poly(vinyl alcohol), PVA, served as efficient photocatalysts for the reduction of CHCl_3_ when swollen in air-saturated solutions of formate buffers were photolyzed with 350 nm photons. The phototransformation generated CH_2_Cl_2_, CO_2_ and Cl^−^ as products. The utilization of the continuous extraction method coupled with in situ potentiometry enabled kinetic determinations of the reaction progress. Quantum yields of halide ion formation, ϕ(Cl^−^), larger than 1 were obtained in the presence of air. These findings, together with the occurrence of a post-irradiation Cl^−^ formation, indicated that the photoreduction took place via a chain process. Reductions photoinitiated by swollen films exhibited ϕ(Cl^−^) values between 3 and 20 times higher than the reactions induced in solutions containing the two polymers. Also, the dependencies of ϕ(Cl^−^) on CHCl_3_ or HCO_2_^−^ concentration diverged significantly from the trends observed using solutions. Most findings are consistent with the occurrence of a reaction mechanism involving SPEEK radicals, •CO_2_^−^ and •CHCl_2_ as chain carriers.

## 1. Introduction

Photosensitive organic polymers are important materials that can drive chemical transformations, employing electromagnetic radiation as the source of energy. These macromolecules, frequently called photopolymers, photocatalysts or photosensitizers have been the subject of recent interest because of their potential ability to initiate a variety of reactions by means of sunlight. Furthermore, they can be frequently bonded chemically to substrates, which enable facile separation of photogenerated products from the macromolecular sensitizer. The simplest synthetic route involves binding molecular chromophores to conventional macromolecular structures, enabling the preparation of materials responding to different regions of the solar spectrum [1]. Another strategy involves the use of conjugated polymers as photocatalysts [2,3]. The approach of binding chromophores to polymers has proven to be quite versatile, including the preparation of photopolymers designed for specific tasks [4,5]. An example of this procedure is the synthesis of polymers containing benzophenone (BP) as a chromophore, which initiates numerous photoreactions including curing, cross-linking and redox processes [4,6,7,8]. BP is a sturdy ketone exhibiting a broad absorption centered at about 350 nm, and excitation of the ketone in the presence of H-atom donors yields free radicals that induce such transformations [9].

The sodium salt of sulfonated poly(ether etherketone), SPEEK, is a macromolecular analogue of benzophenone possessing a BP functionality on each monomer unit. Mixtures (blends) of SPEEK and poly(vinyl alcohol), PVA, mimic the photochemical properties of the BP/2-propanol solution system, which under illumination generates α-hydroxy (ketyl) radicals of BP, (ϕ)_2_C•OH, where ϕ = phenyl group, with a high efficiency [9]. In the polymer system, SPEEK acts as the sensitizer, whereas PVA serves as the H-atom donor. The illumination of SPEEK/PVA blends yields α-hydroxy radicals of the polyketone, HSPEEK•, a strong reducing agent able to dehalogenate several chloromethanes such as CCl_4_, CHCl_3_ and CCl_3_F [10,11,12]. Since both SPEEK and PVA are soluble in H_2_O, the preparation of cross-linked thin polymer films (insoluble in water) is feasible via solution casting. Thus, photoreductions initiated by SPEEK/PVA blends can be studied in water and also using films which are either dry or swollen in aqueous solutions. Recent investigations showed that CCl_4_ and CHCl_3_ undergo efficient chain photoreductions upon the photolysis of air-free solutions of SPEEK/PVA blends in the presence of formate buffers containing HCO_2_H and HCO_2_^−^ [11,12]. CCl_4_ is a toxic pollutant that has been found to be a significant contributor to underground solvent plumes called dense nonaqueous phase liquid (DNAPL) [13]. Chloroform is present as a minor component in DNAPL [14], and a product of CCl_4_-reductive dehalogenation [10]. The illumination of SPEEK in the presence of formate buffers yields •CO_2_^−^ [11], a strong reductant that is an efficient chain carrier.

Currently, the utilization of solar light as a source of renewable energy is of significant interest, but reactions initiated by sunlight are inevitably limited by the periodic day/night cycle. The fact that the photoreductions of CCl_4_ and CHCl_3_ by SPEEK systems proceed via chain processes is interesting since such transformations occur with high quantum yields and continued to operate after illumination ceased [10,11]. Thus, these chain photoreductions offer a possible avenue to circumvent the limitation imposed by the intermittent availability of sunlight. A popular strategy to achieve the dehalogenation of chlorocarbons utilizes dispersions of TiO_2_ particles as photoinitiators [15]. In the case of CHCl_3_, most studies have focused on achieving the elimination of the chlorocarbon from contaminated water. The photodegradation of CHCl_3_ induced by titania particles usually occurs via an oxidation pathway, which proceeds with low efficiencies, in part due to the low chlorocarbon concentration present in relevant samples [16].

In solutions saturated with Ar, the photoreduction of CCl_4_ was 7–11 times more efficient than that of chloroform [10]. However, a chain photoreduction of CHCl_3_ occurred in air-saturated solutions as well [11], but no such process took place for CCl_4_ in the presence of O_2_. Furthermore, the CHCl_3_ photoreduction under air was only 30% less efficient than in the presence of Ar, and proceeded via a different mechanism. In contrast, the photoreduction of CCl_3_F showed a higher efficiency with air than without, but no chain reduction occurred [12]. Such findings were unexpected because O_2_ inhibits transformations involving reducing radicals. Efforts were, therefore, made to identify conditions conducive to improving the efficiency of CHCl_3_ photoreduction in the presence of air. Earlier studies on the O_2_ photoreduction to H_2_O_2_ demonstrated that the quantum yields of peroxide formation were ten times higher when the reaction was initiated using swollen polymer films instead of solutions of the macromolecules [17]. The aim of the present study was to establish whether an enhancement of the chain CHCl_3_ photoreduction under air was achievable when swollen SPEEK/PVA initiated the transformation.

As shown previously, CHCl_3_ was reduced most effectively when formate buffers were present during photolysis [11]. In addition, the reaction efficiency was dependent on the source of the PEEK precursor utilized for the synthesis of SPEEK. The highest quantum yields of photoreduction were obtained with SPEEK made from the precursor provided by Solvay. Consequently, the present report is based on results from experiments that employed SPEEK derived from this precursor. Also, high concentrations of the H-atom donor and excess chloromethanes were previously identified as conditions yielding the highest efficiencies [10,11,12]. Thus, irradiations were conducted under these conditions and using thin, cross-linked SPEEK/PVA films swollen in solutions of formate buffers saturated with air. The kinetic data demonstrated that the photoreduction of CHCl_3_ in the presence of air took place via a chain reaction that was several times more efficient when initiated by swollen SPEEK/PVA films instead of polymer solutions.

## 2. Materials and Methods

### 2.1. Materials

PVA with an average molar mass of M_n_ = 8.9–9.8 × 10^4^ g/mol^−1^ (99% hydrolyzed) and CHCl_3_ were purchased from Sigma-Aldrich, St. Louis, MO 68178, USA. The chloromethane was washed several times with water to remove the stabilizer and stored in the dark. PEEK powder Ketaspire KT-880 FP, M_n_ = 4 × 10^4^ g mol^−1^, was provided as a gift by Solvay. VWR was the source for all other chemicals; preparation of aqueous solutions utilized water purified using a Millipore Milli-Q Biocel system. Sulfonation of PEEK was carried out with H_2_SO_4_ and subsequent transformation into the Na^+^ salt (SPEEK), as well as film cross-linking, which was carried out as described before [17]. Films containing 17/83 wt% SPEEK/PVA were used because in preliminary tests they showed improved reproducibility and efficiency compared with those containing 30% polyketone, which has been employed previously [17]. Typical dimensions of dry films were 5 cm × 2.5 cm and a thickness of 50 (±6) μm. Swelling films in aqueous solutions increased in each dimension by a factor of 1.3; film aging was suppressed via storing them at 4 °C.

### 2.2. Methods

In situ determinations allowed the monitoring of progress of the photoreactions by means of the continuous extraction method [18]. This procedure was utilized previously to characterize the kinetics of H_2_O_2_ photogeneration using swollen SPEEK/PVA films [17]. The earlier experiments verified that a fast equilibration was established between fluid phase and films when the latter were immersed in vigorously stirred solutions. Preliminary tests using ion chromatography (IC) confirmed that Cl^−^ ions photogenerated inside films experienced constant extraction into the aqueous phase, where they were quantified using an ion-selective electrode (ISE), as reported previously [10,11,12].

**Figure 1:**
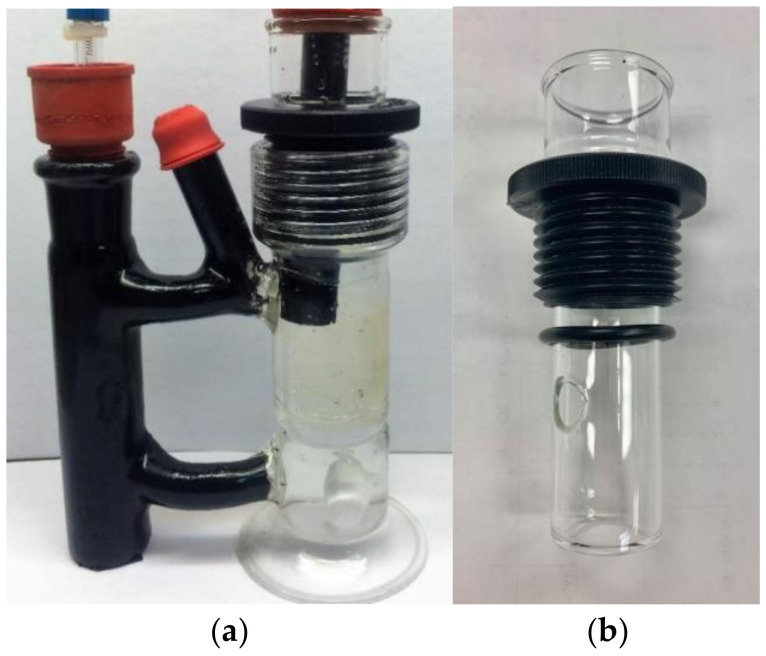
(**a**) Image of the vessel, including ISE, reference electrode and swollen SPEEK/PVA film around the inner tube. (**b**) Image of inner glass tube, including o-ring and nylon bushing cap.

Illuminations were carried out in borosilicate glass vessels similar to the those used earlier for potentiometric determinations [10,11,12], but modified to maintain films fully extended throughout the exposure to light [17]. The modified photoreactor consisted of two vertically positioned tubular glass compartments connected via two short (3.5 cm length, 1.1 cm diameter) horizontal glass tubes. The larger, wider compartment (11.5 cm length, 4 cm diameter) housing the ISE featured a glass base on the bottom and a glass thread adaptor on top. This compartment also housed an inner and vertically positioned glass tube (11 cm long, 3 cm wide, with the top 2 cm wider, and 2 mm thick) that prevented the films from rolling. A circular aperture on the bottom of this tube facilitated fast circulation of the solution. Swollen films were positioned around the outer surface of the vertical glass tube, which was secured inside the larger compartment by means of FETFE o-ring and a nylon bushing cap screwed into the thread adaptor. The narrower compartment (11 cm long and 3 cm wide) included a top glass joint, and housed the light-sensitive reference electrode that was shielded from light. Fused on the top connecting tube was another glass tube (4 cm length and 1 cm diameter) that served as a port for injecting CHCl_3_ or for bubbling gases into the solution through a septum. Both compartments were sealed with perforated septa through which the tightly fitted electrodes were introduced in the solution. A version of this photoreactor modified with a water jacket and connected to a Fisher Isotemp 9000 bath circulator was utilized for post-irradiation experiments conducted at 19 °C. Images of the photoreaction are presented in Figure 1; a diagram of the film location within the vessel can be found in the SI section of reference [17].

Prior to photolysis, 54 mL of the solutions was equilibrated in the dark under continuous stirring in sealed photochemical reactors. For experiments conducted without air, the solutions were bubbled with the desired gas for 20 min under stirring. Unless otherwise indicated, in most experiments 2 mL of CHCl_3_ were injected into the solutions; a large fraction of the chloromethane remained as phase-separated droplets given that the solubility of this compound in water is 6.6 × 10^−2^ M, or 0.3 mL in 54 mL [19]. Illuminations used 350 (±15 nm) photons generated using a circular Rayonet 100 source; the temperature inside the cavity of the illuminator was 29 °C. Except in few cases, all other determinations were performed at room temperature. Determinations of the light intensity (I_0_) employed the Aberchrome 540 actinometer [20]. In situ potentiometric determinations of [Cl^−^] employed an ISE from Thermo Scientific, Waltham, MA 02451, USA, and a Radiometer K601 mercurous sulfate reference electrode in conjunction with a Mettler Toledo SevenMulti S80 dual pH/ion meter. 

Formate buffers, [HCO_2_H] + [HCO_2_^−^] = 0.1–0.5 M, served as H-atom donors and also to maintain a constant ionic strength as required for potentiometric measurements. In experiments without HCO_2_^−^, this was achieved using a salt (NaClO_4_ or NaCH_3_CO_2_) with a concentration of 4 × 10^−3^ M. Most experiments were performed at least twice; deviations in the rates amounting to about 20% were routinely observed, which is typical for the heterogeneous polymer systems [17]. Because a headspace existed above the solutions, illuminations conducted with air-saturated systems yielded the same results when the photoreactor was open or sealed with septa. Detection of additional products using samples withdrawn from sealed photoreactors containing the HCO_2_^−^ solution at pH = 7.3, SPEEK/PVA films and 2 mL of CHCl_3_ after photolysis under air for 2 h. Gas chromatography/mass spectrometry (GC-MS) determinations utilized an Agilent 6890N chromatograph equipped with a Restek Rxi-624Sil MS column (30 m, 0.25 mm ID, 1.4 µm df), coupled to an Agilent 6890N MS detector. The injection port temperature was 220 °C, 5 µL of a headspace sample was injected in split mode, ratio of 20:1, under a He flow of 1.4 mL/min; the GC oven temperature was ramped from 30 to 300 °C at 50 °C/min. CO_2_ determinations in the presence of water vapor are feasible by means of FTIR detection of the characteristic signal at 2349 cm^−1^ [21]. Measurements used a Shimadzu IR-Prestige-21 apparatus and a 10 cm gas cell with KBr windows. A few μL of the solution were injected in the cell purged with N_2_; scans were run after solvent evaporation. Liquid samples also served for IC measurements by means of a Shimadzu Prominence ion chromatograph equipped with a Dionex AS22 column, a Dionex AERS500 ion suppressor and a Shimadzu CCD10A conductivity detector. Analysis occurred in the isocratic mode, the eluent consisted of 4 mM Na_2_CO_3_ and 1.5 mM NaHCO_3_.

## 3. Results

### 3.1. Photochemical Experiments

**Figure 2:**
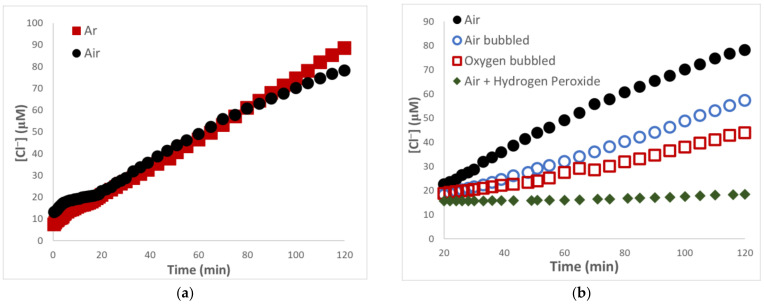
Generation of Cl^−^ during photolysis of SPEEK/PVA films swollen in 0.36 M formate buffer at pH = 7.3 with 2 mL CHCl_3_ exposed to 350 nm photons under different gases, I_0_ = 2.2 × 10^−^^6^ M hv. (**a**) ■ Ar and ● air; (**b**) ● air, ○ air bubbled, ☐ O_2_ and ◆ air + 0.1 mM H_2_O_2_.

Illuminations performed with swollen films in the absence of CHCl_3_ failed to yield Cl^−^ ions; the same result was obtained from control experiments without light. In contrast, Figure 2 shows that chloride ions were generated upon the irradiation of SPEEK/PVA films immersed in the formate buffer at pH = 7.3 exposed to different gases. Displayed in Figure 2a is a comparison of the [Cl^−^] evolution for illuminations in the presence of Ar and air. In air-free systems, the Cl^−^ formation was slow and erratic during the first 10 min of irradiation, and the kinetic data were not reproducible. This period of time is known as the induction period and has been noticed in prior solution photoreductions of halomethanes [10,11,12]. Reproducible kinetic results were obtained once the induction period ceased, at which point [Cl^−^] increased linearly with time for the duration of the illumination. In air-containing solutions, the induction period lasted for about 20 min of photolysis and also yielded minuscule and erratic changes in [Cl^−^]. At longer times, the photogeneration of halide ions was a linear function of time until about 80 min, but turned sublinear beyond that time. The occurrence of induction periods in systems involving reducing radicals originates from the partial scavenging of the reductants by O_2_ to form species unable to reduce halomethanes. The length of the induction period is related to the [O_2_]; extensive discussions about the relationship between induction period and [O_2_] for illuminated SPEEK/PVA blends have been presented previously [10,11].

Earlier studies on the photoreduction of O_2_ by SPEEK/PVA systems indicated that mechanistic information gathered from solution experiments was useful to rationalize the processes initiated by swollen films [17]. In a similar way, the understanding of the current film results can be aided using the findings from the solution photoreduction of CHCl_3_ [11]. At a first sight, comparison of the data shown in Figure 2a with those from solution experiments seem indicate that Cl^−^ formation was slower for the film photoreaction. However, such a comparison is misleading because in the fluid system the reducing species were generated throughout the solution, while in the present study radical formation was confined to the much smaller film volume (~0.14 mL). As will be shown later, meaningful comparisons can be made only on the basis of quantum efficiencies. On the other hand, longer induction periods were detected in film experiments where the rate of radical formation was lower, since less photons were absorbed by the polymeric sensitizer. Furthermore, the film system involved the equilibration of O_2_ via gas transport between three phases: the swollen polymer blend, the solution and the headspace. In contrast, a faster O_2_ equilibration can be expected for the solution systems involving only liquid and gas phases.

Portrayed in Figure 2b are results gathered during several experiments in which O_2_ was present; the data obtained with air are also included to facilitate comparisons. All kinetic runs were performed at pH = 7.3 given that, as will be shown later, the reaction rate was highest at this acidity. An induction period persisting for about 20 min was always observed, but the kinetic data were not included in Figure 2b because of their lack of reproducibility. Increases in [Cl^−^] according to linear functions of time were observed after the induction period in all cases. Numerous photoreactions initiated by SPEEK/PVA blends exhibit zero-order rate laws of product formation [10,11,12,17]. Such a kinetic feature is characteristic of photoreactions where the rate-determining step is the radical formation process, which is controlled by the constant photon flux entering the system. The reaction rate, r = d[Cl^−^]/dt, can be obtained from the slope of the linear [Cl^−^] increase. However, chloride ions generated inside the films experience subsequent dilution upon diffusion into the solution bulk. Thus, the reaction rate corrected for dilution (r_c_) was used to evaluate the quantum yield of Cl^−^ generation, ϕ(Cl^−^) = r_c_/I_0_. The calculation of ϕ(Cl^−^) also requires a correction for I_0_, given that actinometry measures the number of photons entering the vessel, but only a small fraction of the photon flux is absorbed by the film. No simple procedure to correct I_0_ is available due, in part, to the highly nonsymmetrical shape of the photoreactor. Hence, the quantum yields reported here represent only the lower limits of the photoreaction efficiency.

According to the results of Figure 2, the photoreduction was faster in the presence of Ar, with ϕ(Cl^−^) = 2.2, but the efficiency dropped to 1.71 under air. These findings clearly demonstrate that the CHCl_3_ photoreduction took place via a chain process, even when air was present. ϕ(Cl^−^) decreased to 1.2 upon the continuous bubbling of the swelling solution with air, and an even lower efficiency of 0.95 resulted in the presence of pure O_2_. The lowest efficiency (0.19) was obtained in experiments where both air and 0.1 mM H_2_O_2_ were present. The photolysis of films swollen in air-saturated solutions of ClO_4_^−^ yielded ϕ(Cl^−^) = 0.2, which reflects the lower efficiency of HSPEEK• formation when HCO_2_^−^ was absent. PVA was the only H-atom donor present in this system and the resulted polyol radical seemed to be a rather inefficient reductant. Substitution of ClO_4_^−^ by CH_3_CO_2_^−^ doubled the ϕ(Cl^−^) value, suggesting that acetate is a better H-atom donor than PVA. Illumination of SPEEK/PVA air-saturated solutions without the formate buffer induced reduction of O_2_ with a quantum yield of H_2_O_2_ formation equal to 0.02, but the efficiency increased 10 times when swollen films initiated the photoreaction [17].

**Figure 3:**
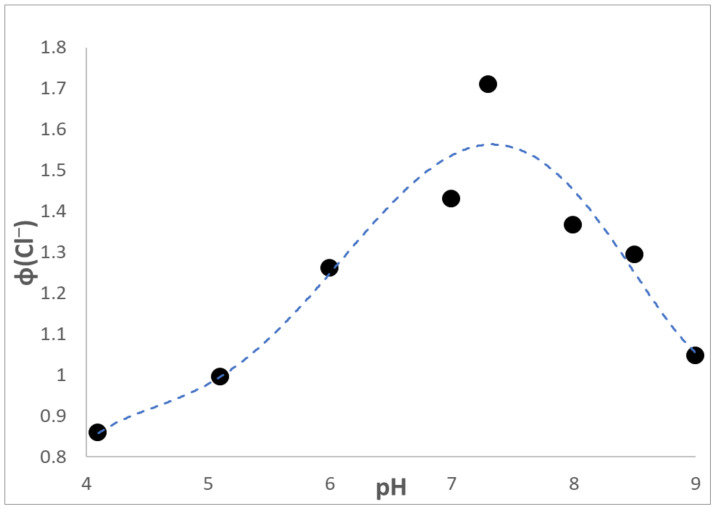
Evolution of ϕ(Cl^−^) a function of pH during illumination of SPEEK/PVA films swollen in a 0.36 M formate buffer saturated with air, containing 2 mL CHCl_3_ and exposed to 350 nm photons wit I_0_ = 2.2 × 10^−^^6^ M hv.

Shown in Figure 3 is the dependence of ϕ(Cl^−^) on the pH of the swelling solution containing air. Results on the efficiency of the CHCl_3_ photoreduction versus pH initiated by solutions of SPEEK/PVA were obtained in the absence of air [11]. Thus, only qualitative comparisons between the present data and the earlier results are meaningful. As in the solution investigation, Figure 3 shows that ϕ(Cl^−^) reached a maximum value at pH = 7.3 and progressively declined with increasing acidity, and also raising basicity, of the swelling solutions. However, less sharp decreases in ϕ(Cl^−^) were noticed during the film-initiated CHCl_3_ photoreduction. According to the data of Figure 3, ϕ(Cl^−^) at pH = 9 was 61% of the maximum efficiency and 50% at pH = 4. In contrast, the solution value at pH = 9 was 33% of the maximum yield, and no CHCl_3_ photoreduction took place at pH = 4 [11]. The large ϕ(Cl^−^) differences between the values determined with films and solutions clearly show that more efficient reductions took place in the former systems. In chain processes propagation steps control the efficiency of the transformations [22]. This means that swollen SPEEK/PVA films provided an environment in which chain propagation steps were more effective than in the polymer solutions.

**Figure 4:**
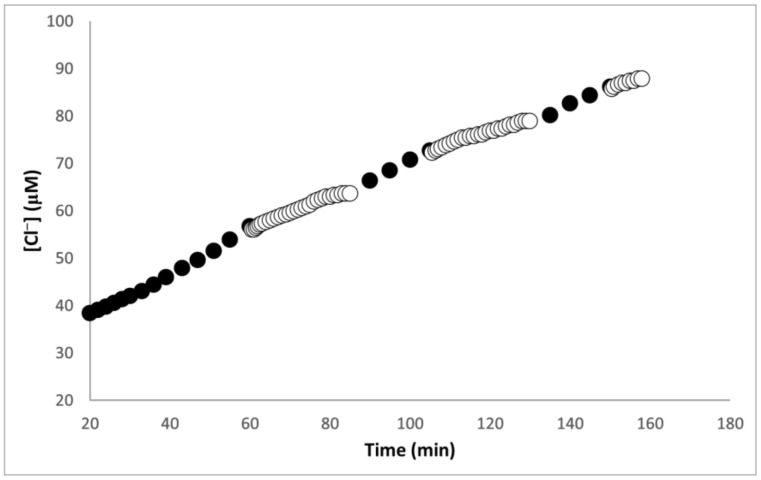
Changes in [Cl^−^] during illumination (●) and dark (○) periods for a SPEEK/PVA film swollen in an air-saturated solution at pH = 7.3 with 0.36 M formate buffer and 2 mL CHCl_3_. T = 19 °C, 350 nm photons, I_0_ = 2.2 × 10^−^^6^ M hv.

Presented in Figure 4 are results from a post-irradiation experiment which was conducted at a constant temperature of 19 °C. This procedure was adopted to avoid the temperature changes that result when the Rayonet illuminator is turned on and off. The initial illumination step lasted 60 min after the induction period. That photolytic step was followed by alternating periods of non-exposure (25 min) and exposure (20 min) to light. Interestingly, [Cl^−^] continued to rise in a fairly linear fashion during the dark periods. Initially, the slope of the linear [Cl^−^] increase without light was only slightly lower than that obtained under photolysis, but the rate of dark reaction decreased at longer times. While linear increases in [Cl^−^] were also noticed during post-irradiation experiments in air-saturated polymer solutions [11], the rates were more than 10 times lower in the dark than those measured under illuminations. In contrast, for films, the rates of Cl^−^ formation in the dark were about 87% of the photolytic values. Since the rates of the dark reaction reflect the efficiency of propagation steps [22], the findings of Figure 4 support the idea that cross-linked films provide conditions favoring chain processes.

**Figure 5:**
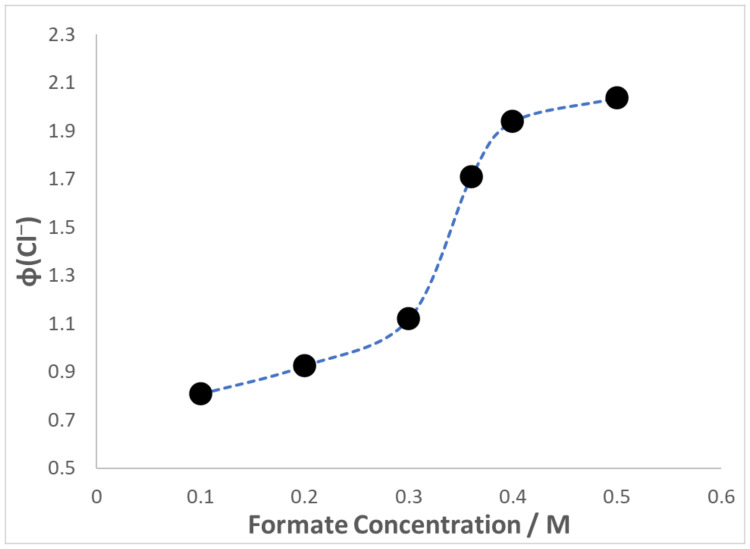
Dependence of ϕ(Cl^−^) on the concentration of formate buffer during illumination with 350 nm photons of SPEEK/PVA films swollen in air-saturated solutions at pH = 7.3 that also contained 2 mL CHCl_3_; I_0_ = 2.2 × 10^−^^6^ M hv.

Figure 5 depicts the change in efficiency of chloride ion generation at pH = 7.3 for films swollen in solutions containing increasing amounts of formate buffer. Efficient chain reductions require the presence of high concentrations of both substrate and H-atom donor. The reason for this is that both substrates and reducing agents participate in chain propagation steps and increases in their concentration increase the speed of the propagations. The optimum conditions for the occurrence of chain photoreactions were identified during the CCl_4_ reduction in SPEEK/PVA solutions [10]. A maximum ϕ(Cl^−^) value was noted for solutions containing 0.36 M HCO_2_^−^, and subsequent investigations employed this optimized concentration. However, the results of Figure 5 indicate a steady increase in ϕ(Cl^−^) with rising [formate]. This dependence is logical given that experiments without formate clearly demonstrated that efficient chain reductions took place only in the presence of this H-atom donor.

**Figure 6:**
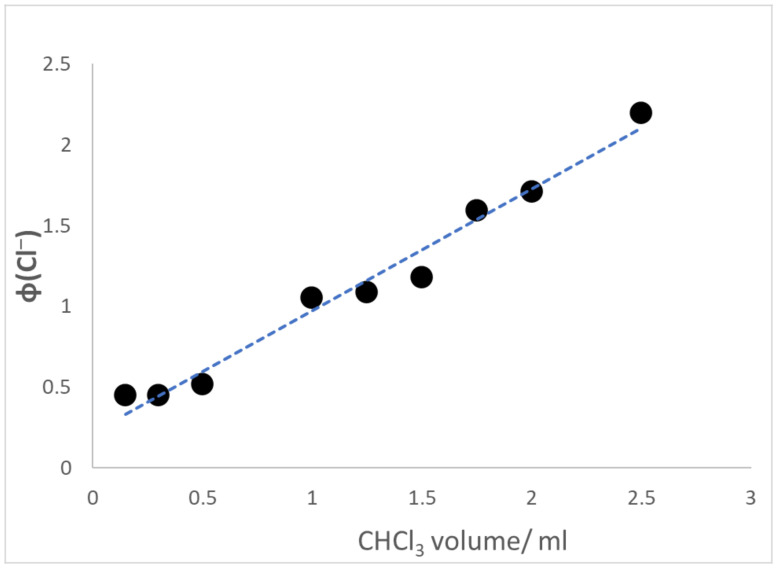
Variation of ϕ(Cl^−^) as a function of the CHCl_3_ volume added to the air-saturated formate buffer at pH = 7.3 used to swell the SPEEK/PVA films. Illuminations employed 350 nm photons with I_0_ = 2.2 × 10^−^^6^ M hv. The solubility limit of CHCl_3_ corresponds to 0.3 mL of chloromethane.

Illustrated in Figure 6 is the dependence of the quantum yield on the amount of CHCl_3_ present in the swelling solution. The data of Figure 6 indicate that ϕ(Cl^−^) remained constant at [CHCl_3_] below or equal to the solubility limit. However, a large and linear increase in ϕ(Cl^−^) ensued upon the introduction of excess halomethane. These findings differ vastly from the behavior of the CHCl_3_ photoreduction in SPEEK/PVA solutions [11]. For the fluid system, ϕ(Cl^−^) increased linearly at [CHCl_3_] ≤ 6.6 × 10^−2^ M (the solubility limit), and only small additional increases in efficiency occurred when excess halomethane was added. Obviously, the findings shown in Figure 6 imply that phase-segregated CHCl_3_ was able to contribute significantly to the photoreduction process.

### 3.2. Additional Determinations

GC/MS analysis was performed using headspace samples withdrawn from sealed photoreactors after illumination. CH_2_Cl_2_ was only detected after the induction period. FTIR determinations confirmed the formation of CO_2_; oxalate ions were identified as products by means of IC. Efforts to detect H_2_O_2_ or CO were unsuccessful.

## 4. Discussion

The data presented in Figure 2 indicate that SPEEK/PVA are efficient materials for photoinitiating the CHCl_3_ reduction in air-saturated solutions. As demonstrated in an earlier solution study, the reduction involves photochemically generated HSPEEK• [11]. Solution reactions of free radicals are frequently limited due to consumption of these species via radical–radical processes, and reductions involving HSPEEK• behave similarly [17]. The decay of α-hydroxy radicals derived from small molecules is usually diffusion-controlled; in the case of (ϕ)_2_C•OH (a model for HSPEEK•), the second-order rate constant is k = 8.5 × 10^8^ M^−1^ s ^−1^ [23]. However, the bulky radical of PVA decays through a complex dimerization/disproportionation process with a rate “constant” that decreases as the reaction unfolds [24]. Radicals of polyelectrolytes, such as those of ionized poly(acrylic acid), persist for hours in air-free solutions as their mobility is further decreased by interchain electrostatic repulsions [25]. While SPEEK is a polyelectrolyte, HSPEEK• decays fast in solution but very slowly in cross-linked films [17]. H_2_O_2_ photogeneration induced by swollen films is 10 times more efficient than in solutions because cross-links present in the solid matrix hinder the diffusion of HSPEEK•. Consequently, in films, higher numbers of radicals can participate in the O_2_ reduction. Thus, an enhancement in ϕ(Cl^−^) was anticipated for swollen films where reactions between HSPEEK• radicals are less efficient than in the fluid medium. In the solution study on the CHCl_3_ photoreduction, only experiments conducted under Ar and air utilized SPEEK derived from the Solvay precursor [11]. Hence, a direct comparison of quantum efficiencies is only meaningful for those experiments. According to the data, ϕ(Cl^−^) was ≥ 3 times higher for photoreductions initiated by swollen films. The fact that ϕ(Cl^−^) values larger than 1 were obtained clearly indicates that the reduction of CHCl_3_ involved a chain process.

The results from Figure 2b indicate that the photoreduction was less efficient when the swelling solution was saturated with O_2_ instead of air. Considering that for air-saturated water [O_2_] = 0.26 mM and 1.3 mM upon saturation with oxygen [26], the data imply that the reaction efficiency decreased with increasing oxygen concentration in the swelling solution. The same trend was noticed when the CHCl_3_ photoreduction was induced in SPEEK/PVA solutions [11]. Regarding these experiments, the present ϕ(Cl^−^) values are 10–20 times higher than those obtained with the PEEK precursor that eventually produced less reactive HSPEEK•. The results of Figure 2 clearly indicate that the photogenerated reducing radicals reacted with O_2_. Since no H_2_O_2_ was detected during the photoreaction, a logical conclusion is that any formed peroxide was further reduced to water. Hence, the reduction of O_2_ consumed four reducing radicals, which became unavailable to reduce CHCl_3_. This rationalization seems helpful to explain the decrease in ϕ(Cl^−^) induced when both air and H_2_O_2_ were present. The scavenging of reducing radicals by the peroxide yields OH^-^ and OH• [27], but the latter reacts fast with HCO_2_^−^, reforming CO_2_^−^. This means that H_2_O_2_ induced a retardation effect, because any HSPEEK• radical that reacted with the peroxide was not lost but just diverted from reducing CHCl_3_.

As shown in Figure 3, the efficiency of the CHCl_3_ photoreduction dropped both in acidic and basic solutions. Such behavior suggests that the photogeneration of HSPEEK• was affected by the pH of the swelling solution. HSPEEK• is formed through H-atom abstraction using an excited state of SPEEK. For BP, the (*n*, π*) triplet excited state (the precursor of (ϕ)_2_C•OH) is quenched efficiently by H_3_O^+^, k_q_ = 6 × 10^8^ M^−1^ s^−1^ [28]. Hence, the decrease in ϕ(Cl^−^) with increasing acidity can be rationalized if an analogous quenching of the SPEEK excited state took place. On the other hand, both OH^−^ and Cl^−^ quench the triplet BP excited state, albeit with lower quenching constants, k_q_ = 5 × 10^6^ M^−1^ s^−1^ and 2.2 × 10^5^ M^−1^ s^−1^, respectively [29]. This means that the gradual decrease in ϕ(Cl^−^) above pH = 7.3 may have resulted from the combined quenching of the SPEEK excited state by the anions. Overall, the quantum efficiencies for the photoreaction initiated by films were at least >3 times the values obtained by illumination of SPEEK/PVA solution. Hence, the swollen SPEEK/PVA films provided an environment that enhanced the chain photoreduction of CHCl_3_ at all pH values.

Earlier solution investigations showed that post-irradiation experiments can provide independent evidence of a chain process [10,11,12]. The data of Figure 4 demonstrated that Cl^−^ generation continued after photolysis was interrupted. In contrast, post-illumination product formation was not observed during the photoreduction of CCl_3_F and O_2_ by SPEEK/PVA blends, because no chain process was involved in these transformations [12,17]. Hence, the results of Figure 4 confirm that a chain process operated during the CHCl_3_ photoreduction.

Both the low ϕ(Cl^−^) values obtained when the formate buffer was absent, and the data shown in Figure 5 provide evidence that efficient photoreduction of CHCl_3_ occurred only when HCO_2_^−^ was available to serve as H-atom donor. Note that the line included in the plot is a guide to the eye only; no specific relationship between ϕ(Cl^−^) and [formate] is implied. Since SPEEK is a polyelectrolyte containing SO_3_^-^ and Na^+^ counterions, swollen SPEEK/PVA films behave in a fashion similar to cation-exchange membranes [30]. This means that partitioning of HCO_2_^−^ into the films was conditioned by the formate concentration present in the swelling solution. According to this interpretation, the results of Figure 5 reflect the extent of HCO_2_^−^ incorporation into the swollen polymer films.

The findings summarized in Figure 6 indicate that the efficiency of the photoreduction was low in solutions containing chlorocarbon amounts equal or lower than the solubility limit of CHCl_3_ in water. Thus, CHCl_3_ molecules dissolved in the swelling solution contributed only moderately to the photoreduction. The most important contributors to the chain process were phase-separated small halomethane droplets. Sub-micron-sized CHCl_3_ droplets are anticipated to have migrated into the swollen films, where they became accessible to radicals acting as chair carriers. Given that HSPEEK• is a polyelectrolyte radical, this species experiences counterion condensation, particularly at the high ionic strength imposed by the formate buffer [31]. Hence, inside the film, HSPEEK• probably exists as an ion-paired neutral species able to reduce CHCl_3_ molecules present in the droplets. Increasing the excess CHCl_3_ in the swelling solution results (under stirring) in an augmentation of the number of droplets present, which explains the unusual dependence of ϕ(Cl^−^) on the volume of the halomethane presented in Figure 6.

The combined findings of the present study demonstrate that the photoreduction of CHCl_3_ in the presence of air occurred efficiently when initiated by swollen SPEEK/PVA films. In addition, higher yields were determined using swollen films instead of polymer solutions. The detection of Cl^−^, CH_2_Cl_2_ and CO_2_ as main products indicates that the photoreaction can be accounted for on the basis of the chain mechanism proposed for the solution study [11]. This mechanism was extensively analyzed before and is not included in this report. A short summary of the mechanism is useful to rationalize the present results. In this mechanism, HSPEEK• and •CO_2_^−^ are generated via H-atom abstraction from HCO_2_^−^ by the excited state of SPEEK. CHCl_3_ reduction by HSPEEK• yields Cl^−^ and •CHCl_2_, which then abstracts an H-atom from formate, producing CH_2_Cl_2_ and reforming •CO_2_^−^. Because [SPEEK] >> [CHCl_3_], the generated •CO_2_^−^ is anticipated to react preferentially with the polyketone instead of CHCl_3_, reforming HSPEEK• plus CO_2_. In this mechanism HSPEEK•, •CO_2_^−^ and •CHCl_2_ are the chain carriers, while chain termination proceeds via radical–radical reactions. The radical–radical reaction of •CO_2_^−^ generates oxalate ions [27], and their detection as products adds further support to the proposed mechanism.

In air-containing systems, O_2_ competes with CHCl_3_ for HSPEEK•:

HSPEEK• + O_2_ → SPEEK + HO_2_• (1)

HSPEEK• + CHCl_3_ → SPEEK + •CHCl_2_ + Cl^−^ + H^+^ (2)

While the rate constant for these processes is not known, (CH_3_)_2_C•OH can serve as a model of HSPEEK•. The rate constant for the reduction of O_2_ by (CH_3_)_2_C•OH is 2 × 10^9^ M^−1^ s ^−1^, whereas the value for the reaction with CH_2_Cl_2_ amounts to k ≈ 1 × 10^6^ M^−1^ s ^−1^ [27]. Although the rate constant for the reduction of CHCl_3_ by (CH_3_)_2_C•OH is unknown, the value for the reaction of the radical with CH_2_Cl_2_ can serve as a rough approximation. Using the concentrations of both chemicals in the swelling solution enables the estimation of the pseudo-first-order rate constants, k_1_ = 5.2 10^5^ s ^−1^ and k_2_ = 6.6 × 10^4^ s ^−1^. These rough estimates indicate that step 1 predominates until [O_2_] decreases significantly. They also mean that the lengthy induction period preceding Cl^−^ formation originates from preferential HSPEEK• consumption via step 1 until a low steady state [O_2_] is reached. The k_2_ value is certainly a lower limit, given that the calculation employed the solubility limit of CHCl_3_. According to the results shown in Figure 6, the CHCl_3_ concentration inside the films is higher than that value.

## 5. Conclusions

The findings of the present investigation demonstrated that SPEEK/PVA films can operate as efficient photocatalysts for the reduction of CHCl_3_ in the presence of air. In fact, the quantum yields determined under such conditions were higher than those measured in solutions without O_2_. A secondary reaction pathway for the transformation of CHCl_3_ was identified in the previous solution study yielding CO as a product [11], which means that a complete dehalogenation of the halocarbon took place. However, the available evidence indicates that this interesting reaction channel was not viable in the film system. Nevertheless, the utilization of cross-linked SPEEK/PVA films yields not only enhanced efficiencies, but also enables the easy separation of the products from the swollen solid photocatalysts. The findings from experiments performed at different pH values, and also those from post-irradiation measurements, suggest that the films provide an environment that facilitates chain propagations’ steps.

## Data Availability

Not applicable.

## References

[1] Ferguson C.T.J., Zhang K.A.I. (2021). Classical Polymers as Highly Tunable and Designable Heterogeneous Photocatalysts. ACS Catal..

[2] Banerjee T., Podjaski F., Kröger J., Biswal B.P., Lotsch B.V. (2021). Polymer Photocatalysis for Solar-to-Chemical Energy Conversion. Nat. Rev. Mater..

[3] Dai C., Liu B. (2020). Conjugated Polymers for Visible Light-Driven Photocatalysis. Energy Environ. Sci..

[4] Zhou J., Allonas X., Ibrahim A., Liu X. (2019). Progress in the Development of Polymeric and Multifunctional Photoinitiators. Prog. Polym. Sci..

[5] Nowakowska M., Szczubialka K. (2017). Photoactive Polymeric and Hybrid Systems for Photocatalytic Degradation of Water Pollutants. Polym. Degrad. Stab..

[6] Yang P., Yang W. (2013). Surface Chemoselective Phototransformation of C-H Bonds on Organic Polymeric Materials and Related High-Tech Applications. Chem. Rev..

[7] Koizume H., Shiraishi Y., Hirai T. (2008). Temperature-Controlled Photosensitization Properties of Benzophenone-Conjugated Thermoresponsive Copolymers. J. Phys. Chem. B.

[8] Bourdelande J.L., Font J., Sánchez-Ferrando F. (1983). The Use of Insoluble Benzoylated Polystyrene Beads (Polymeric Benzophenone) in Photochemical Reactions. Can. J. Chem..

[9] Gilbert A., Baggot J. (1991). Essentials of Molecular Photochemistry.

[10] Black J.R., Islam M.S., Carmichael H.L., Slaten B.L., Little B.K., Mills G. (2017). Radical Chain Reduction of CCl_4_ Initiated by Illumination of SPEEK Solutions. J. Phys. Chem. A.

[11] Islam M.S., Duin E.C., Slaten B.L., Mills G. (2018). Photoreduction of CHCl_3_ in Aqueous SPEEK/HCO_2_^-^ Solutions Involving Free Radicals. J. Phys. Chem. A.

[12] Islam M.S., Dissanayaka R., Higgins B.T., Adhikari S., Mills G. (2019). Photoreduction of CCl_3_F in Aqueous Solutions Containing Sulfonated Poly(ether etherketone) and Formate Buffers. Res. Chem. Intermed..

[13] Kueper B.H., Stroo H.F., Vogel C.M., Ward C.H., Kueper B.H., Stroo H.F., Vogel C.M., Ward C.H. (2014). Source Zone Remediation: The State of the Practice. Chlorinated Solvent Source Remediation.

[14] Rubin H., Hillel D. (2005). POLLUTION/Groundwater. Encyclopedia of Soils in the Environment.

[15] Henderson M.A. (2011). A Surface Science Perspective on TiO_2_ Photocatalysis. Surface Sci. Rep..

[16] Hsiung T.-L., Wei L.-W., Huang H.-L., Wang H.P. (2021). In situ X-ray Absorption Spectroscopy Studies of TiO_2_ Photocatalytic Active Sites for Degradation of Trace CHCl_3_ in Drinking Water. J. Synchrotron Rad..

[17] Lockhart P., Little B.K., Slaten B.L., Mills G. (2016). Photogeneration of H_2_O_2_ in Water-Swollen SPEEK/PVA Polymer Films. J. Phys. Chem. A.

[18] Henson J.H.L., Hybart F.J. (1972). The Degradation of Poly(vinyl Chloride). I. Hydrogen Chloride Evolved from Solid Samples and from Solutions. J. Appl. Polym. Sci..

[19] Horvath A.L. (1982). Halogenated Hydrocarbons: Solubility-Miscibility with Water.

[20] Heller H.G., Langan J.R. (1981). Photochromic heterocyclic fulgides. Part 3. The use of (E)-α-(2,5-dimethyl-3-furylethylidene)(isopropylidene)succinic anhydride as a simple convenient chemical actinometer. J. Chem. Soc. Perkin Trans. 2.

[21] Griffith D.W.T., Deutscher M.N., Caldow C.G.R., Kettlewell G., Riggenbach M., Hammer S. (2012). A Fourier Transform Trace Gas Analyzer for Atmospheric Applications. Atmos. Meas. Tech. Discuss..

[22] Huyser E.S. (1970). Free-Radical Chain Reactions.

[23] Ledger M.B., Porter G. (1972). Primary Photochemical Processes in Aromatic Molecules. Part 15.—The Photochemistry of Aromatic Carbonyl Compounds in Aqueous Solution. J. Chem. Soc. Faraday Trans. 1.

[24] Ulanski P., Bothe K., Rosiak J.M., von Sonntag C. (1994). OH-Induced Crosslinking and Strand Breaking of Poly(vinyl alcohol) in Aqueous Solution in the Absence and Presence of Oxygen. A Pulse Radiolysis and Product Study. Macromol. Chem. Phys..

[25] Ulanski P., Bothe E., Hildenbrand K., Rosiak J.M., von Sonntag C. (1996). Hydroxyl-Radical-Induced Reactions of Poly(Acrylic Acid); a Pulse Radiolysis, EPR and Product Study. Part I. Deoxygenated Aqueous Solutions. J. Chem. Soc. Perkin Trans. 2.

[26] Tromans D. (1998). Temperature and Pressure Dependent Solubility of Oxygen in Water: A Thermodynamic Analysis. Hydrometallurgy.

[27] Neta P., Grodkowski J., Ross A.B. (1996). Rate Constants for Reactions of Aliphatic Carbon-Centered Radicals in Aqueous Solution. J. Phys. Chem. Ref. Data.

[28] Ramseier M., Senn P., Wirz J. (2003). Photohydration Benzophenone in Aqueous Acid. J. Phys. Chem. A.

[29] Shizuka H., Obuchi H. (1982). Anion-Induced Triplet Quenching of Aromatic Ketones by Nanosecond Laser Photolysis. J. Phys. Chem..

[30] Kitto D., Kamcev J. (2022). Mannig Condensation in Ion Exchange Membranes: A Review on Ion Partitioning and Diffusion Models. J. Polym. Sci..

[31] Liu S., Ghosh K., Muthukumar M. (2003). Polyelectrolyte Solutions with Added Salt: A Simulation Study. J. Chem. Phys..

